# Optimal Concentration and Duration of Endotracheal Tube Coating to Achieve Optimal Antimicrobial Efficacy and Safety Balance: An In Vitro Study

**DOI:** 10.3390/gels9050414

**Published:** 2023-05-16

**Authors:** Manar Fathy Al-Sayed, Mohamed Tarek El-Wakad, Mohammed A. Hassan, Ahmed M. Soliman, Amal S. Eldesoky

**Affiliations:** 1 Department of Biomedical Engineering, Faculty of Engineering, Helwan University, Cairo 11511, Egypt; mohammed.ali@h-eng.helwan.edu.eg (M.A.H.); ahmed_soliman05@h-eng.helwan.edu.eg (A.M.S.); 2 Department of Biomedical Engineering, Higher Technological Institute, Cairo 11511, Egypt; amal-eldesoky70@hotmail.com; 3 Faculty of Engineering & Technology, Future University, Cairo 11835, Egypt; mohamed.elwakad@fue.edu.eg

**Keywords:** endotracheal intubation, mechanical ventilation, ventilator-associated pneumonia, antimicrobial agents, endotracheal tube, antibiotics, antifungals, antiseptics, silver nitrate sol-gel, biocompatible, antimicrobial efficacy, toxicity, bacteria proliferation

## Abstract

Background: Ventilator-associated pneumonia (VAP) is a common and genuine complication in fundamentally sick patients accepting mechanical ventilation. Silver nitrate sol-gel (SN) has been proposed as a potential preventative measure against VAP. Be that as it may, the arrangement of SN with distinctive concentrations and pH values remains a basic factor influencing its effectiveness. Methods: Silver nitrate sol-gel was arranged with distinctive concentrations (0.1852%, 0.03496%, 0.1852%, and 0.01968%) and pH values (8.5, 7.0, 8.0, and 5.0) separately. The antimicrobial action of the silver nitrate and NaOH arrangements were assessed against *Escherichia coli* as a reference strain. The thickness and pH of the arrangements were measured, and biocompatibility tests were performed on the coating tube. The auxiliary changes in the endotracheal tube (ETT) tests after treatment were analyzed utilizing electron microscopy (SEM) and transmission electron microscopy (TEM). Results: The pH estimations of the diverse arrangements showed that the pH values shifted depending on the test conditions, with pH values extending from 5.0 to 8.5. The consistency estimations of the arrangements showed that the thickness values expanded as the pH values drew closer to 7.5 and diminished when the pH values went over 7.5. The antimicrobial action of the silver nitrate and NaOH arrangements were successful against *Escherichia coli*, with microbial checks decreasing in concentration (0.03496%, 0.1852% (pH: 8), and 0.01968%). The biocompatibility tests revealed tall cell reasonability rates, demonstrating that the coating tube was secure for therapeutic utilization and did not hurt typical cells. The SEM and TEM investigation gave visual proof of the antibacterial impacts of the silver nitrate and NaOH arrangements on the bacterial surface or interior of the bacterial cells. Moreover, the investigation revealed that a concentration of 0.03496% was the foremost successful in hindering the development of ETT bacterial colonization at the nanoscale level. Conclusions: We propose that cautious control and alteration of the pH and thickness of the arrangements are essential to guaranteeing the reproducibility and quality of the sol-gel materials. The silver nitrate and NaOH arrangements may serve as a potential preventative degree against VAP in sick patients, with a concentration of 0.03496% appearing to show the most elevated viability. The coating tube may serve as a secure and viable preventative measure against VAP in sick patients. Further investigation is required to optimize the concentration and introduction time of the arrangements to maximize their adequacy in avoiding VAP in real-world clinical settings.

## 1. Introduction

Pneumonia is the second most common type of nosocomial infection after urinary tract infection and the most common problem in mechanically ventilated patients in the intensive care unit. The length of intubation increases the risk of disease. Hospitalized patients on mechanical ventilation by endotracheal tube (ETT) intubation are at increased risk of lung infection from hospitalized bacteria [1,2,3,4,5,6,7].

Bacterial colonization of the endotracheal tubes (ETTs) is a common problem in critically ill patients and can lead to ventilator-associated pneumonia (VAP) [8]. Silver nitrate (AgNO_3_) is a water-soluble salt that releases silver ions into a solution, which have broad-spectrum activity against bacteria and can kill them and prevent their colonization [9,10,11,12]. Therefore, the use of silver nitrate has been studied for its potential to prevent VAP and bacterial colonization of ETTs [13,14].

Several studies have investigated the use of silver-coated or silver-nitrate-treated ETTs to prevent VAP and bacterial colonization. Studies have demonstrated that the use of silver-coated or silver-nitrate-treated ETTs can significantly reduce the incidence of VAP and the duration of mechanical ventilation compared to standard ETTs [10,15,16].

Silver nanoparticles, particularly those synthesized using silver nitrate (AgNO_3_), have shown promising antimicrobial activity against a broad spectrum of bacterial strains. The mode of action of AgNO_3_ nanoparticles against bacteria is believed to be due to their ability to disrupt the integrity of the bacterial cell wall, leading to cell death. The nanoparticles may be attached to the bacterial cell wall, where they can cause leakage of cytoplasmic contents and damage to the membrane. Additionally, AgNO_3_ nanoparticles can penetrate the bacterial cell and interfere with its metabolic processes. This includes inhibiting DNA replication, protein synthesis, and enzyme activity, ultimately leading to bacterial death. The ability of AgNO_3_ nanoparticles to target bacterial cells makes them a promising candidate for the development of novel antibacterial agents. However, further research is needed to determine the optimal conditions for their use and potential side effects on human health and the environment [17,18,19,20].

A study by Kollel et al. (2008) investigated the use of silver-coated ETTs to prevent VAP in mechanically ventilated patients. The study found that using silver-plated ETTs significantly reduced the incidence of VAP and the duration of mechanical ventilation compared to standard ETTs. Another study by Krupali et al. (2013) applied silver nitrate to the inner surface of ETT to prevent bacterial colonization. The study found that the administration of silver nitrate significantly reduced the incidence of bacterial colonization and VAP in critically ill patients [9,15,21].

A systematic review by Li Bassi et al. (2015) evaluated the use of different strategies to prevent infections associated with ETT. The review found that the use of silver ETT and the application of silver nitrate were effective in reducing the incidence of VAP and bacterial colonization [22,23,24].

However, some studies have raised concerns about the potential toxicity of silver nitrate. A study by Maki et al. (2006) found that silver nitrate was associated with an increased risk of skin irritation and chemical burns in patients receiving silver-nitrate-coated catheters [25,26,27,28,29].

The use of silver nitrate with endotracheal tubes appears to be an effective strategy to prevent bacterial colonization and VAP in critically ill patients. However, the potential toxicity of silver nitrate should be carefully considered before use. The optimal concentration and duration of silver nitrate administration for endotracheal intubation (ETT) remain undetermined, and there is no consensus on the exact dosage concentration and time of use [25,30].

Some studies have used concentrations of 0.5% to 1% silver nitrate solution applied to the inner surface of the ETT for 30 min to 2 h, while others have used lower concentrations (0.1% to 0.3%). A study by Krupali et al. (2013) used a 1% silver nitrate solution, which was applied to ETT for 30 min, and found a significant reduction in bacterial colonization and VAP in critically ill patients. Another study by Pacheco-Fowler et al. (2009) used a lower concentration (0.1%) for a longer period (24 h) and found that it was also effective in reducing bacterial colonization [9,30,31,32,33,34,35,36].

It is important to note that the optimal concentration and duration of silver nitrate administration may vary depending on the type of ETT, the patient population, and the clinical setting. The potential toxicity of silver nitrate should also be considered when determining the appropriate concentration and duration of application [28,29,31,34,35,36].

However, the potential toxicity of silver nitrate is a concern; silver nitrate is a chemical compound that can potentially cause harm to the human body through various mechanisms of damage. One possible way that silver nitrate can enter the human body is through direct skin contact, which can cause skin irritation, burns, and discoloration. This is particularly true for concentrated solutions of silver nitrate, which can be highly corrosive to the skin and other tissues [25,30,34,37,38].

Another way that silver nitrate can enter the human body is through inhalation of silver nitrate fumes or dust particles. This can cause irritation and damage to the respiratory system, leading to coughing, wheezing, and shortness of breath. Long-term exposure to silver nitrate fumes or dust can also lead to chronic respiratory problems, such as bronchitis and asthma.

Ingestion of silver nitrate is also a possible route of exposure, particularly in cases where silver nitrate is accidentally ingested or intentionally consumed as a form of self-harm. Ingestion of silver nitrate can cause severe damage to the digestive system, including abdominal pain, vomiting, and bloody diarrhea. In extreme cases, ingestion of silver nitrate can lead to acute poisoning, which can be fatal if not treated promptly [39,40,41,42].

The mechanisms of damage caused by silver nitrate are complex and can involve a range of factors, including the concentration of the solution, the duration of exposure, and the route of exposure. To fully understand the potential harm of silver nitrate, it is important to analyze the various ways in which it can enter the human body and the specific mechanisms of damage that it can cause. Therefore, determining the optimal concentration and duration of silver nitrate administration for ETTs is critical to achieving an optimal balance between antimicrobial efficacy and safety [37,43,44,45,46,47,48].

In summary, the use of silver nitrate with ETTs appears to be an effective strategy to prevent VAP and bacterial colonization in critically ill patients. However, the potential toxicity of silver nitrate should be carefully considered when determining the appropriate concentration and duration of application to achieve an optimal balance between antimicrobial efficacy and safety [14,23,49,50,51,52,53]. Pneumonia is a common nosocomial infection in hospitalized patients, particularly those who are mechanically ventilated via endotracheal intubation. Bacterial colonization of ETTs is a prevalent problem in critically ill patients and can lead to the development of VAP [54,55,56]. Therefore, preventing VAP is a critical goal in the management of critically ill patients. The use of silver nitrate has been studied for its potential to prevent VAP and bacterial colonization of ETTs, and several studies have demonstrated its effectiveness. However, further research is needed to determine the optimal concentration and duration of silver nitrate administration for ETTs in different patient populations and clinical settings [1,3,57,58,59].

In conclusion, the application of silver nitrate in endotracheal tubes (ETTs) demonstrates effectiveness in preventing (VAP) and bacterial colonization in critically ill patients. Nevertheless, the potential risk of silver nitrate toxicity necessitates careful consideration of the concentration and duration of application. Additionally, the cost-effectiveness of this intervention and the potential development of bacterial resistance should be considered. Therefore, the use of silver nitrate in ETTs should be implemented as a component of a comprehensive infection prevention strategy for critically ill patients [14,23,49,50,51,52,53].

This article identifies the optimal concentration and duration of ETT silver nitrate administration to achieve an optimal antimicrobial efficacy and safety balance [59,60,61,62,63,64,65,66,67,68,69].

## 2. Results and Discussion

SN sol-gel was prepared with four different concentrations. pH was measured for each preparation. ETT tubes were cut and immersed three times each period for three days.

### 2.1. pH Measurement

To measure the pH of each solution used in the experiments, a digital pH meter was employed. The pH values of the solutions were recorded and tabulated in Table 1. The results of the pH measurements showed that the pH values of the solutions varied depending on the experimental conditions. In experiment (1), the pH value of the solution was measured at 8.5, while in experiment (2), the pH value was 7. In experiment (3), the pH value was measured at 8, and in experiment (4), the pH value was 5. These pH measurements are crucial for determining the effects of the different experimental conditions on the solutions’ pH and how they may have impacted the performance of the silver nitrate and NaOH solutions in preventing bacterial colonization and VAP in the ETT samples.

**Figure 1:**
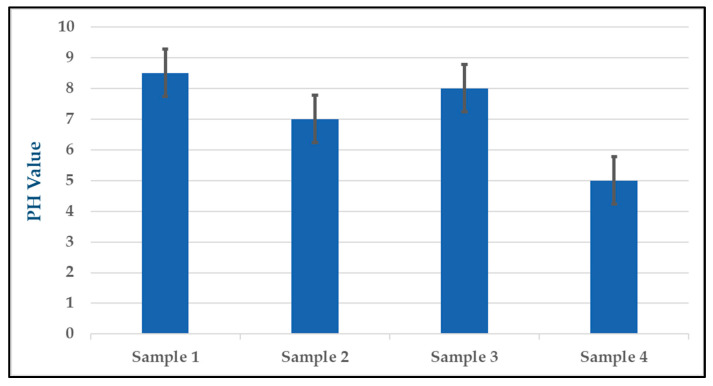
Error analysis for pH values of four samples.

The pH of the sol-gel solution is an important parameter that can affect the sol-gel reaction rate, the stability of the resulting gel, and the properties of the final product. Therefore, careful control and adjustment of the pH is necessary to ensure the reproducibility and quality of the sol-gel materials. To better visualize the variability of the pH values in the four samples, a graph was created with standard error bars for each data point as shown in Figure 1. The standard error bars for each data point convey the degree of variability in the pH measurements and help to illustrate the precision and consistency of the data. Overall, the pH measurements provide important information about the chemical properties of the solutions used in the experiments and help to support the conclusions drawn from the antimicrobial performance data.

### 2.2. Viscosity Measurement

**Figure 2:**
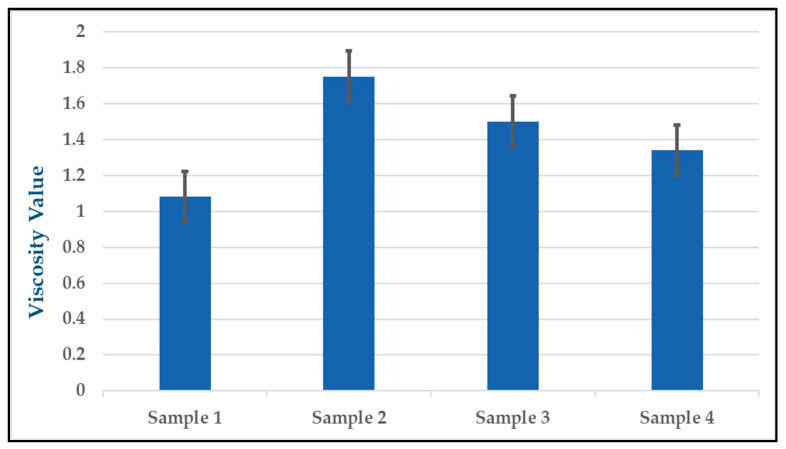
Error analysis for viscosity values of four samples.

To assess the physical properties of the solutions used in the experiments, a viscometer was employed to measure the viscosity of each solution. The results of the viscosity measurements are shown in Table 2, which indicates that the viscosity values of the solutions varied depending on the experimental conditions. The viscosity value of the solution in the experiment (1) was measured at 1.84, while in the experiment (2) it was 1.75. In sample (3), the viscosity value was 1.50, and in sample (4), it was 1.34. The results of the viscosity measurements showed that the viscosity values increased as the pH values of the solutions approached 7.5, after which they decreased as the pH values went above 7.5, which is consistent with previous research [63,64]. We emphasize the need for further experiments with pH above 7.5, with only one group meeting this conclusion in the viscosity measurements. The viscosity measurements are important for understanding the properties of the solutions and how they may have impacted the effectiveness of the silver nitrate and NaOH solutions in preventing bacterial colonization and VAP in the ETT samples. By measuring the viscosity values of the different solutions, it is possible to understand any changes in the thickness or flow of the solution and how these changes may have affected the antimicrobial properties of the solutions. These results can be visualized through a graph with standard error bars for the viscosity measurements, shown in Figure 2. The standard error bars for each data point convey the degree of variability in the viscosity measurements and help to illustrate the precision and consistency of the data. The graph provides a more complete picture of the experimental results and helps to support the conclusions drawn from the antimicrobial performance data.

### 2.3. Tested Microorganisms

To evaluate the antimicrobial efficacy of various solutions, a Gram-negative bacterium was used to measure the mean zone of inhibition in millimeters. The concentrated samples were assessed at the Antimicrobial Activity Unit of Al-Azhar University’s Regional Center for Mycology and Biotechnology, and the outcomes are shown in Table 3.

In sample (1), the tested microorganisms produced a positive result, indicating that the solution did not inhibit bacterial growth (the initial microbial count of *Escherichia coli* was 12 CFU/µL). Conversely, in samples (2), (3), and (4), the tested microorganisms produced a negative result, indicating that the solution had an antibacterial effect, and the microbial count of *Escherichia coli* was reduced to 5, 7, and 3 CFU/µL, respectively. These findings are significant in understanding the effectiveness of silver nitrate and NaOH solutions in preventing bacterial colonization and ventilator-associated pneumonia in endotracheal samples. The results suggest that the solutions may serve as a preventative measure against ventilator-associated pneumonia in critically ill patients. The reliable and accurate results of testing the samples at a specialized antimicrobial activity unit provide confidence in the conclusions regarding the effectiveness of the solutions in inhibiting bacterial growth.

### 2.4. Biocompatibility and Cell Viability

**Figure 3:**
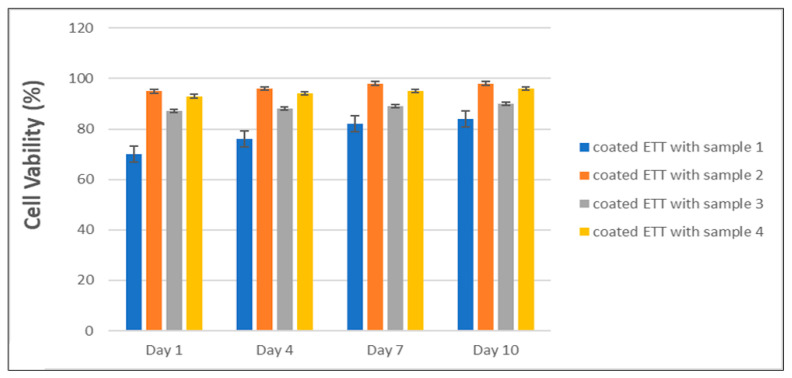
Cell viability of coated ETT with samples (1, 2, 3, and 4).

The results of the biocompatibility tests are significant in demonstrating the safety of the coating tube for medical use and its potential for preventing bacterial colonization and VAP in ETT samples without causing any harm to normal cells [65]. The high cell viability percentages, which were proximately 70%, 97%, 86%, and 95% for samples 1, 2, 3, and 4, respectively, as shown in Figure 3, indicate that the coating tube solution did not have any adverse effects on the normal cells, allowing them to grow and thrive in the presence of the coatings. These results suggest that the coating tube may serve as a safe and effective preventative measure against VAP in critically ill patients. The biocompatibility tests were conducted under controlled conditions, and the validity of the conclusions drawn from the results is supported using reliable and accurate methods.

### 2.5. SEM Measurement

**Figure 4:**
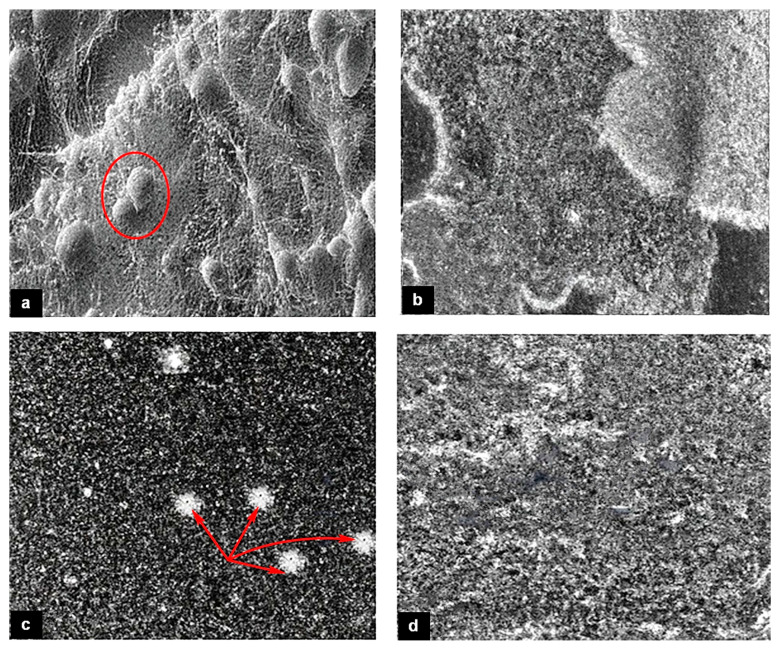
Scanning electron microscope (SEM) images of samples (**a**) sample 1, (**b**) sample 2, (**c**) sample 3, and (**d**) sample 4.

**Figure 5:**
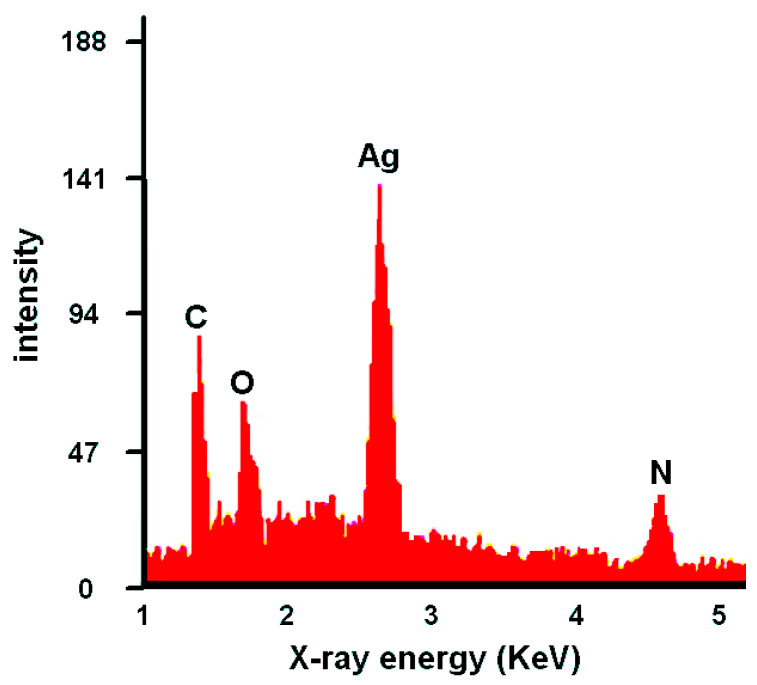
EDX analysis for elements in experiments.

The SEM images of different samples reveal the different sizes, shapes, and distributions of the nanoparticles on the bacterial surface or inside the bacterial cells. Figure 4 exhibits the SEM images to show the morphological microstructure surface of AgNO_3_ nanoparticles that interact with ETT bacteria under different preparation conditions. Images (b) and (d) for samples 2 and 4, respectively, show nearly homogenous surfaces which means uniform limited growth of ETT bacterial colonization. These findings are valuable in supporting the effectiveness of the SN and NaOH solutions in preventing bacterial colonization and VAP in the ETT samples. Images (a) and (c) for samples 1 and 3, respectively, show large bacterial annexation and non-homogeneous surface which means a large growth rate of ETT bacterial colonization. Moreover, the energy-dispersive X-ray (EDX) analysis displays the elemental composition of the nanoparticles and confirms the presence of silver ions and chemical ingredients of ETT bacteria as shown in Figure 5. Generally, the AgNO_3_ nanoparticles may be attached to the bacterial cell wall and disrupt its integrity, causing leakage of cytoplasmic contents and cell death. Alternatively, AgNO_3_ nanoparticles can penetrate bacterial cells and interfere with their metabolic processes, such as DNA replication, protein synthesis, and enzyme activity [17,18,19,20,66]. Therefore, silver or silver-containing composites are considered antibacterial activities.

### 2.6. TEM Measurement

**Figure 6:**
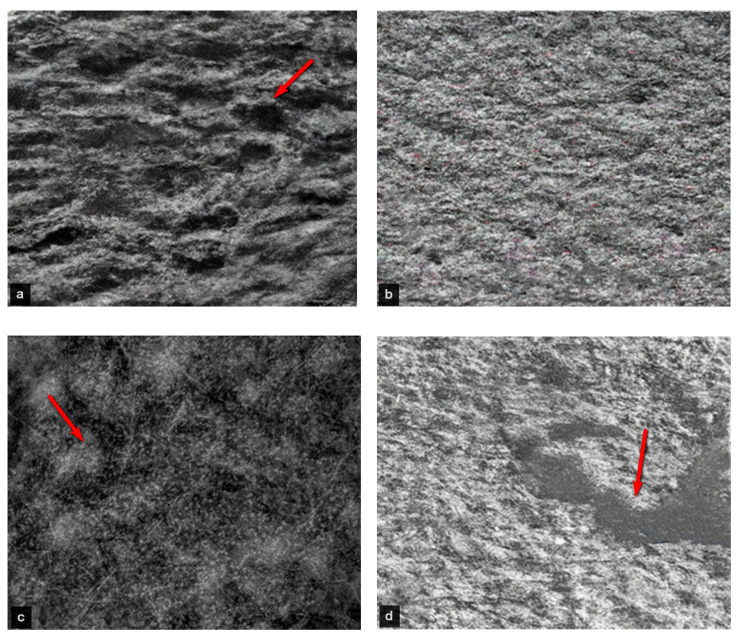
Transmission electron microscopy (TEM) images of samples (**a**) sample 1, (**b**) sample 2, (**c**) sample 3, and (**d**) sample 4.

It is worth noting that the effective antibacterial mechanism of nanosilver nitrate is based on the role of silver and nitrate ions that have antibacterial properties. It can release silver ions that can penetrate the bacterial cell wall and membrane, and interact with proteins and DNA, causing damage and death to the bacteria [68,69,70]. The microstructure features of nano silver nitrate added to ETT bacteria can be seen by using transmission electron microscopy examination, which depends on the shape, size, and distribution of the silver nanoparticles and the ETT bacterial cells. Depending on the several factors mentioned above, e.g., concentration and exposure time of nano silver nitrate, the microstructure may vary in terms of the degree of bacterial damage and aggregation of silver nanoparticles. The surface morphology of the deposited films of the samples is shown in Figure 6. Isolated islands with intermediate spaces can be observed. Irregularly well-organized large, elongated colonies can be observed in Figure 6a for the sample. Smooth bead-like shapes with less agglomeration are distinguished in sample 2 as shown in Figure 6b. The TEM image of sample 3 as shown in Figure 6c exhibits more randomly oriented colonies with coalescence at different regions. With decreasing AgNO_3_ concentration as well as a lower pH value in sample 4, as shown in Figure 6d, a large empty valley region is observed. The surface roughness increases due to the growth in some regions and isolated islands in other regions due to the agglomeration effect. Moreover, the outcomes of the TEM analysis revealed that sample 2 is more effective in inhibiting the growth of ETT bacterial colonization at the nanoscale level.

**Discussion:** The results of the study indicate that the preparation of silver nitrate sol-gel (SN) with different concentrations and pH values is crucial for the effectiveness of the coating tube in preventing bacterial colonization and VAP in endotracheal tube (ETT) samples. The pH measurements of the different solutions showed that the pH values varied depending on the experimental conditions, which could impact the performance of the silver nitrate and NaOH solutions in preventing bacterial colonization and VAP in the ETT samples. The viscosity measurements of the solutions showed that the viscosity values increased as the pH values approached 7.5 and decreased as the pH values went above 7.5. These findings are consistent with previous research and provide valuable information on the properties of the solutions used in the experiments.

The antimicrobial activity of the silver nitrate and NaOH solutions was evaluated using *Escherichia coli* as a reference strain. The results showed that the solutions effectively inhibited bacterial growth, with the microbial count of *Escherichia coli* reduced in samples 2, 3, and 4. These findings suggest that the solutions may serve as a preventative measure against ventilator-associated pneumonia in critically ill patients. The biocompatibility tests of the coating tube revealed high cell viability percentages, indicating that the coating tube did not have any adverse effects on normal cells. This is a significant finding as it suggests that the coating tube may serve as a safe and effective preventative measure against VAP in critically ill patients [12,66,71].

The SEM and TEM analysis provided valuable insights into the structural changes in the ETT samples after treatment, highlighting the effectiveness of the coating tube in preventing bacterial adhesion. The SEM images of the samples revealed the different sizes, shapes, and distributions of the nanoparticles on the bacterial surface or inside the bacterial cells. The TEM images showed the microstructure features of nano silver nitrate added to ETT bacteria and how it may vary depending on the concentration and exposure time of the nano silver nitrate. The analysis also revealed that concentration 0.03496% was the most effective in inhibiting the growth of ETT bacterial colonization at the nanoscale level [12,72].

## 3. Conclusions

In conclusion, the study’s findings suggest that the preparation of silver nitrate sol-gel with different concentrations and pH values is crucial for the effectiveness of the coating tube in preventing bacterial colonization and VAP in ETT samples. The pH and viscosity measurements of the solutions provide valuable information on the properties of the solutions and how they may impact the effectiveness of the coating tube. The antimicrobial activity tests and biocompatibility tests of the coating tube suggest that it may serve as a safe and effective preventative measure against VAP in critically ill patients. The SEM and TEM analysis provided valuable insights into the structural changes in the ETT samples after treatment and highlighted the effectiveness of the coating tube in preventing bacterial adhesion. Future studies are needed to evaluate the effectiveness of the coating tube in clinical settings and its potential for preventing other types of infections.

## 4. Materials and Methods

### 4.1. Material Used

Silver nitrate sol-gel (SN), sodium hydroxide (SH), distilled water (DW), and acetic acid (AA) with molecular weights 169.873, 40.01, 188.015, and 60.052 g/mol, respectively, were supplied by Egyptian chemical manufacturers. ETT tubes cuffed/size 7.5 mm L 28 cm/dimensions 18 × 3 × 0.25 in were supplied by ULTRA ME in Egypt. SN and SH concentrations and stirring times are shown in Table 4.

### 4.2. Silver Nitrate Sol-Gel Preparation

**Figure 7:**
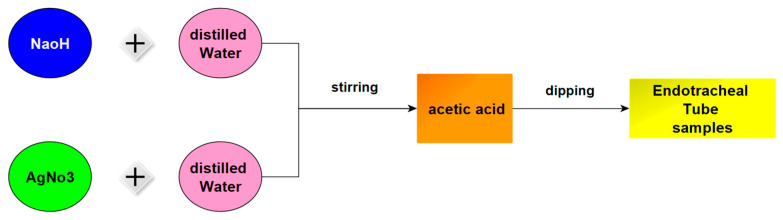
SN sol-gel prepared sequence.

SN sol-gel was prepared with four concentrations, pHs, viscosities, stirring times, and temperatures as shown in Table 4. SN and SH were dissolved in DW and stirred at a specific time and temperature, then AA was added drop by drop and stirred again. Finally, ETT samples were dipped in a solution according to the sequence shown in Figure 1. The dipping samples for samples (1, 2, 3, and 4) are shown in Figure 7, respectively.

**Figure 8:**
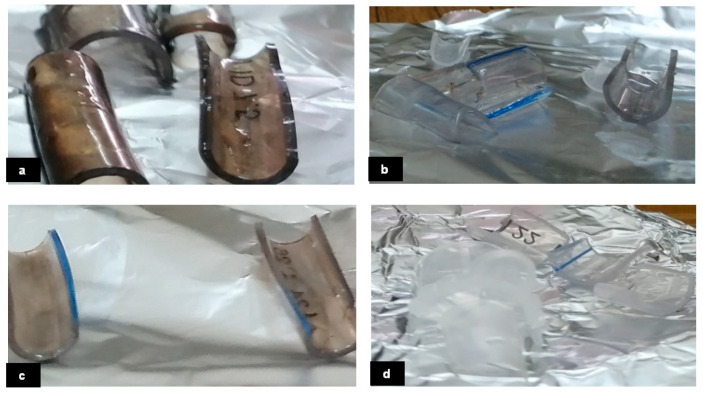
The dipping samples were in sol-gel for 24 h. (**a**) Silver concentration is 0.1852% (pH = 8.5), (**b**) silver concentration is 0.03496% (pH = 7), (**c**) silver concentration is 0.1852% (pH = 8), (**d**) silver concentration is 0.01968% (pH = 5).

In the initial and third trials, a solution of AgNO_3_ and NaOH was prepared by dissolving them in distilled water (DW) and stirring them for sixteen minutes at 45 °C. Then, acetic acid (AA) was added drop by drop, and the mixture was stirred for an additional forty-six minutes. In the second experiment, AgNO_3_ was dissolved in DW, and NaOH was dissolved in DW and stirred for fifty-one minutes at 50 °C. Then, AA was added drop by drop to the solution. In the fourth experiment, AgNO_3_ and NaOH were dissolved in DW and stirred for an hour at 50 °C. AA was then added drop by drop to the solution. In each experiment, four ETT samples were dipped in the solution for 24 h after scraping them to help the mixture stick to them better and faster. After every three hours, the samples were washed with distilled water, dried, and then returned to the solution, as illustrated in Figure 8.

### 4.3. pH Test

A digital pH meter, pH-200, with a measurement range of 2–10 pH units and an accuracy of at least ±0.01 pH units was used to measure the pH of each solution during the experiments. The temperature was maintained at 50 °C and the meter was calibrated using standard buffer solutions before each measurement. Regular pH measurements were recorded to monitor any changes that occurred over time. These pH measurements were important for assessing the impact of different experimental conditions on the solutions’ pH and their effectiveness in preventing bacterial colonization and VAP in the ETT samples [73,74].

### 4.4. Viscosity Test

A digital rotational viscometer (DRV), VSC-E1 Series analyzer, was used to analyze the viscosity of the AgNO_3_ sol-gel experiment. The DRV analyzer was capable of measuring viscosity at shear rates below 10 s^−1^, making it suitable for assessing the performance of the solution for dipping and coating. The analyzer had a small sample size of 0.5 mL and a temperature range of 50 °C. The accuracy of the DRV analyzer was at least ±1%, and the repeatability precision was at least ±0.5%, ensuring precise and reliable measurements. The viscosity of the solution was measured at different time intervals during the experiment to determine the optimal conditions for its performance in preventing bacterial colonization and VAP in the ETT samples.

### 4.5. Antibacterial Effect Measurements

**Figure 9:**
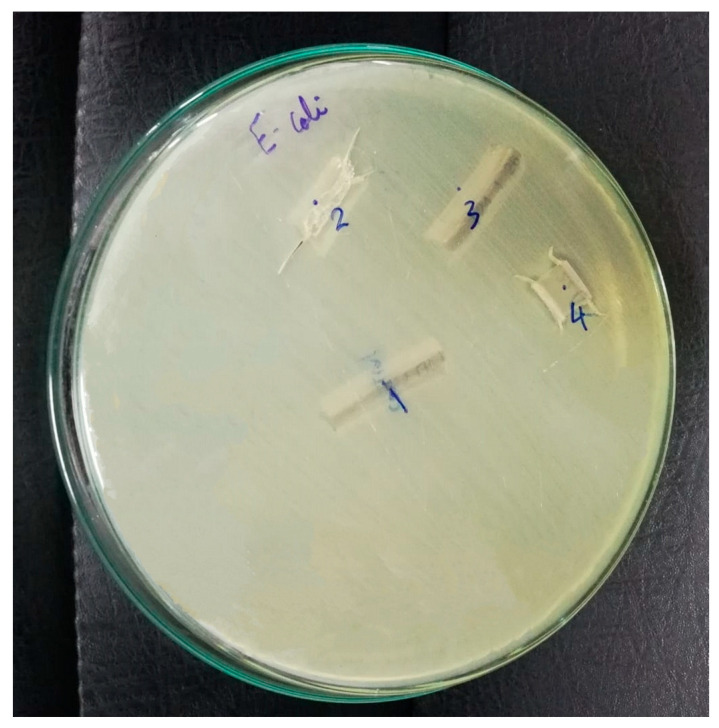
Gram-negative bacteria: *Escherichia coli* ATCC 25922 for all samples.

The study used a Gram-negative bacterium, *Escherichia coli* ATCC 25922 [75,76,77,78], in all samples to evaluate the effectiveness of silver nitrate and NaOH solutions in preventing bacterial colonization in ETT samples as shown in Figure 9. This strain was chosen due to its well-characterized genome sequence, non-pathogenic nature, extensive study in bacterial physiology, and usefulness as a model for studying antibiotic resistance and cellular processes unique to Gram-negative bacteria. Its well-established characteristics and suitability for use as a reference or control strain in experimental studies made it an appropriate choice for the current study.

The study utilized the diffusion agar technique to test the antimicrobial activity of the solutions against the bacterial strain. The technique involved creating a well in the center of an agar plate containing the bacterial culture and adding 100 µL of the solution being tested to the well. Plates were incubated at 37 °C for 24 h, and the inhibition zone diameter was measured to determine antimicrobial activity. The test was repeated using a standardized bacterial strain and consistent testing conditions to ensure accurate and reproducible results. These standardized conditions allowed for comparison and evaluation of the effectiveness of different solutions in preventing bacterial colonization and VAP in the ETT samples.

### 4.6. Biocompatibility Test

To evaluate the biocompatibility of the coating tube with normal cells, cell viability was measured using the trypan blue exclusion assay. Normal cell lines were cultured in RPMI 1640 medium under controlled conditions. To assess cell viability, the cells were treated with the coating tube solution, and the absorbance of the treated cells was measured and compared to that of the control cells. The viability of the cells was calculated as the ratio of the absorbance of the treated sample to that of the control. This method allowed for the evaluation of the coating tube’s biocompatibility with normal cells and any potential cytotoxic effects. The results were important in determining the coating tube’s safety for medical use and potential for preventing bacterial colonization and VAP in ETT samples without harming normal cells.

### 4.7. SEM (Scanning Electron Microscope) Test

SEM analysis is a powerful technique used to obtain high-resolution images of a sample’s surface. The process involves several steps, including sample preparation, shining an electron beam onto the sample, sensing the acquired signal, and processing the resulting image. The SEM (JSM-IT200) instrument settings used in this study were optimized to ensure high-quality images with sufficient resolution. SEM analysis allowed for visualization of the surface morphology of the ETT samples after treatment with different solutions, providing insight into the effectiveness of the coating tube in preventing bacterial colonization and VAP. These images were crucial in determining the coating tube’s potential as a preventative measure against VAP in critically ill patients.

### 4.8. TEM (Transmission Electron Microscopy) Test

TEM examination is a powerful technique used to obtain high-resolution images of the internal structures of samples at the nanoscale. The TEM analysis process involves passing an electron beam through a thin sample, detecting the resulting signal, and creating an image of the structure. TEM settings include an accelerating voltage of 120 kV, a beam current of 200 nA, and a detector for bright-field or dark-field images. TEM analysis allowed for the visualization of the internal structures of the ETT samples after treatment with different solutions, providing insight into the effectiveness of the coating tube in preventing bacterial colonization and VAP. The high-resolution images obtained by TEM provided valuable information about structural changes in the ETT samples after treatment.

## Figures and Tables

**Table 1:** Experiment’s pH results.

Experiment Samples	Silver Nitrate Concentrations	Experiment Duration (Hour)	Temperature (°C)	pH Value
Sample (1)	0.1852%	Twenty-Four	45	8.5
Sample (2)	0.03496%		50	7
Sample (3)	0.1852%		45	8
Sample (4)	0.01968%		50	5

**Table 2:** Experiment’s viscosity results.

Experiment Samples	Viscosity Value
Sample (1)	1.08
Sample (2)	1.75
Sample (3)	1.50
Sample (4)	1.34

**Table 3:** Tested microorganisms’ results.

Experiments Sample	Tested Microorganisms	Microbial Count/Level of Growth Inhibition
Sample (1)	*Escherichia coli*	12 CFU/µL
Sample (2)		3 CFU/µL
Sample (3)		7 CFU/µL
Sample (4)		5 CFU/µL

**Table 4:** Material amount in each experiment.

Experiment Samples	SN Concentrations	SH Concentrations	Stirring Time (Min)
Sample (1)	0.1852%	5	62
Sample (2)	0.03496%	2.6	51
Sample (3)	0.1852%	4	62
Sample (4)	0.01968%	0.400	60

## Data Availability

Not applicable.
